# Epstein-Barr virus glycoprotein gH/gL antibodies complement IgA-viral capsid antigen for diagnosis of nasopharyngeal carcinoma

**DOI:** 10.18632/oncotarget.7688

**Published:** 2016-02-24

**Authors:** Rui-Chen Li, Yong Du, Qiu-Yao Zeng, Lin-Quan Tang, Hua Zhang, Yan Li, Wan-Li Liu, Qian Zhong, Mu-Sheng Zeng, Xiao-Ming Huang

**Affiliations:** ^1^ Department of Otorhinolaryngology Head and Neck Surgery, Sun Yat-Sen Memorial Hospital of Sun Yat-Sen University, Guangzhou, P. R. China; ^2^ State Key Laboratory of Oncology in South China, Collaborative Innovation Center for Cancer Medicine, Sun Yat-sen University Cancer Center, Guangzhou, P. R. China; ^3^ Department of Clinical Laboratory, Sun Yat-Sen University Cancer Center, Guangzhou, P. R. China; ^4^ Department of Nasopharyngeal Carcinoma, Sun Yat-Sen University Cancer Center, Guangzhou, P. R. China; ^5^ Department of Otorhinolaryngology, Guangdong Provincial Hospital of Chinese Medicine, Guangzhou, P. R. China

**Keywords:** nasopharyngeal carcinoma, Epstein-Barr virus, biomarker, gH/gL, viral capsid antigen

## Abstract

To determine whether measuring antibodies against Epstein-Barr virus (EBV) glycoprotein gH/gL in serum could improve diagnostic accuracy in nasopharyngeal carcinoma (NPC) cases, gH/gL expressed in a recombinant baculovirus system was used in an enzyme-linked immunosorbent assay (ELISA) to detect antibodies in two independent cohorts. Binary logistic regression analyses were performed using results from a training cohort (*n* = 406) to establish diagnostic mathematical models, which were validated in a second independent cohort (*n* = 279). Levels of serum gH/gL antibodies were higher in NPC patients than in healthy controls (*p* < 0.001). In the training cohort, the IgA-gH/gL ELISA had a sensitivity of 83.7%, specificity of 82.3% and area under the curve (AUC) of 0.893 (95% CI, 0.862-0.924) for NPC diagnosis. Furthermore, gH/gL maintained diagnostic capacity in IgA-VCA negative NPC patients (sensitivity = 78.1%, specificity = 82.3%, AUC = 0.879 [95% CI, 0.820 - 0.937]). Combining gH/gL and viral capsid antigen (VCA) detection improved diagnostic capacity as compared to individual tests alone in both the training cohort (sensitivity = 88.5%, specificity = 97%, AUC = 0.98 [95% CI, 0.97 - 0.991]), and validation cohort (sensitivity = 91.2%, specificity = 96.5%, AUC = 0.97 [95% CI, 0.951-0.988]). These findings suggest that EBV gH/gL detection complements VCA detection in the diagnosis of NPC and aids in the identification of patients with VCA-negative NPC.

## INTRODUCTION

While nasopharyngeal carcinoma (NPC) is rare in most populations worldwide, the incidence peaks in South China, where NPC occurs in 50 out of every 100,000 individuals [1, 36]. In many of these cases, Epstein-Barr virus (EBV) detection may be useful for detecting NPC and might be a prognostic marker [5]. Elevated IgA antibodies against viral capsid antigen (VCA), early antigen (EA) and nuclear antigens (EBNA1) are outstanding features of such NPC patients [4]. Compared with other antibodies, IgA-VCA is still the most sensitive serological EBV antibody [6], and is detectable even before the development of NPC [4, 37]. Recently, plasma EBV DNA, another biomarker in NPC patients, was used for diagnostic purposes [38] as well as for monitoring NPC patient response to therapy and risk of relapse [7, 8]. Regardless of the high sensitivity and specificity of IgA-VCA in the diagnosis of NPC, IgA-VCA was undetectable in 4 - 24% of patients [6, 9–12], and resulted in misdiagnosis. Quantitative polymerase chain reaction (qPCR) analysis of circulating EBV DNA resulted in tumor detection sensitivities of 22 - 86%, 48 - 95%, 74 - 100% and 79-100% in patients with stage I, II, III and IV disease, respectively [9]. Unlike patients with stage I NPC, which has a 5-year survival rate of 100% after treatment, and stage II, which has a 5-year survival rate of 95% after treatment, the prognosis of patients with stage IV disease is usually poor [9, 13]. Therefore, reliable diagnostic biomarkers to complement IgA-VCA or EBV DNA are required to improve diagnostic accuracy.

EBV infection of epithelial cells requires three viral glycoproteins, gB, gH, and gL, which are conserved among herpesviruses [14, 15]. EBV infection of B cells also requires the viral glycoprotein gp42, which binds the cell-surface major histocompatibility complex (MHC) class II molecules [14, 15]. Furthermore, EBV glycoproteins play an important role in the humoral immune response, and sera against these proteins often neutralize the virus [16]. Therefore, we propose that glycoproteins have the potential to serve as diagnostic biomarkers for NPC.

In the present study, we developed a novel ELISA using a baculovirus-expressed gH (aa 18-679) and gL (aa 24-137) protein complex to test the feasibility of antibodies against gH/gL in the detection of patients with NPC. We compared the sensitivities and specificities of these antibodies separately and in combination with IgA-VCA or circulating EBV DNA in the detection of NPC.

## RESULTS

### Production and purification of soluble EBV gH/gL protein

The stability and proper folding of gH requires gL. Based on preliminary experiments (data not shown), cells were infected with the baculovirus at an MOI of 5 and harvested 72 h post-infection. After purification with Ni chelate-Sepharose chromatography, proteins were quantified and purity was evaluated. SDS/PAGE gels revealed two major bands of 85 kDa and 26 kDa, which corresponded to the recombinant gH and gL proteins, respectively (Figure S1A). Immunoblotting with an anti-flag monoclonal antibody revealed specific bands of the same size (Figure S1B), which suggests that the soluble gH/gL proteins are the major components of the purified proteins. The observed molecular masses corresponded well to the combination of theoretical amino acid sequences and putative N-linked glycosylation. Thus, the purified proteins were considered to have specific antigenicity and could be used in a subsequent ELISA.

### Elevated levels of gH/gL antibodies in NPC patients

To determine whether gH/gL could be applied for NPC diagnosis, we developed an ELISA to detect anti-gH/gL antibodies in sera from a training cohort (*n* = 208 patients with NPC and 198 healthy controls). A comparison of NPC patients with healthy controls (Table S1) showed that only family history (*p* < 0.001) was significant. Age (*p* = 0.973), sex (*p* = 0.388) and smoking history (*p* = 0.622) were not significant.

**Figure 1:**
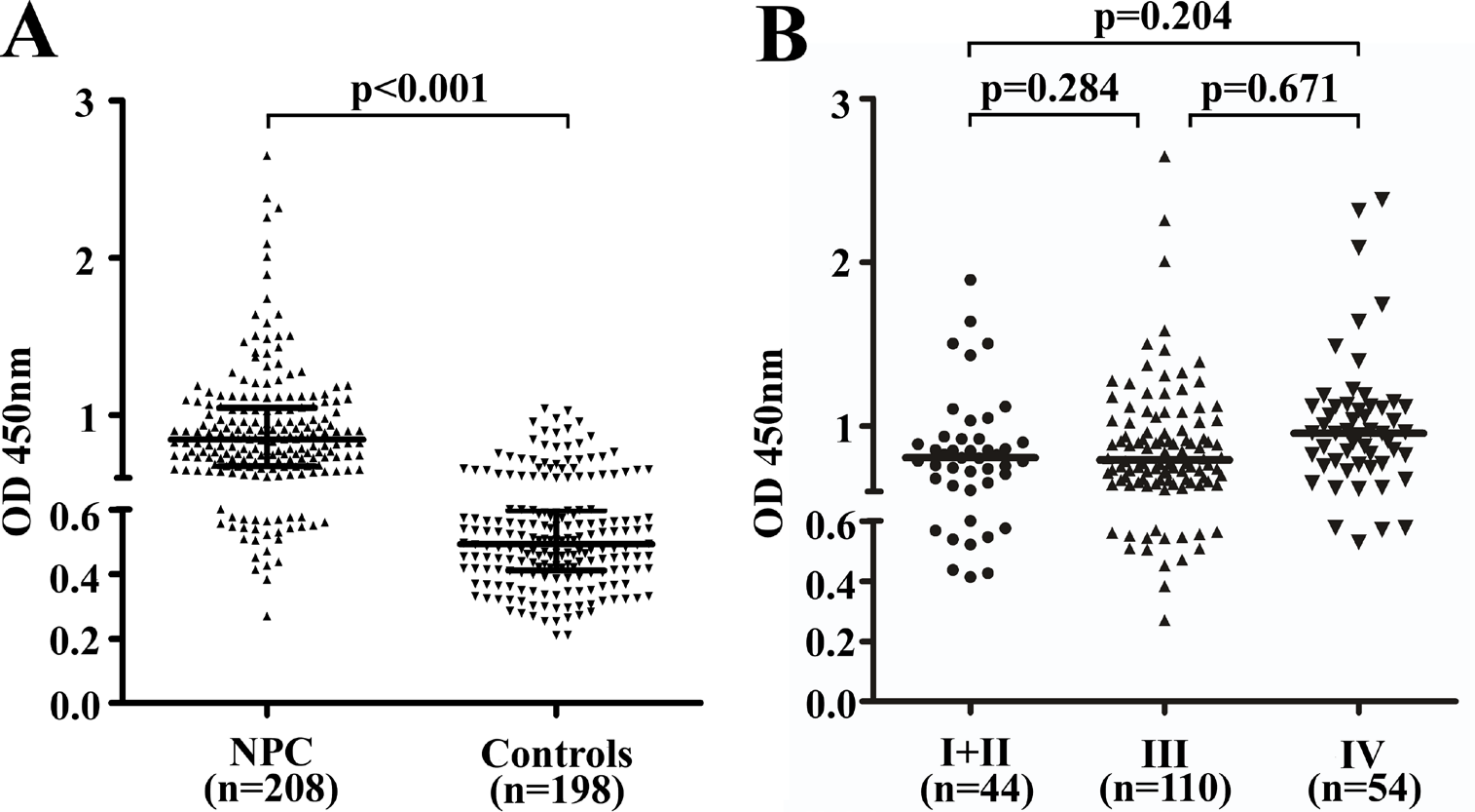
Characteristics and diagnostic values of IgA-gH/gL in the training cohort Scatter plots of the distribution of IgA-gH/gL ELISA results for NPC cases (*n* = 208) and healthy controls (*n* = 198) **A.** Black horizontal lines are medians. The upper black horizontal line indicates the 75th percentile of the data set, and the lower line indicates the 25th percentile. ELISA results for pretreatment serum of NPC patients with stage I (*n* = 10), II (*n* = 34), III (*n* = 110) or IV (*n* = 54) disease **B.** Black horizontal lines are medians. The IgA-gH/gL OD value distributions were not significantly different between stages.

We evaluated the distribution of blood IgA antibodies against EBV gH/gL in patients using OD values (median ± IQR [IQR, interquartile range]). Antibody titers against gH/gL were elevated in a majority of patients with NPC compared to controls (Figure 1A). The median gH/gL OD value for NPC patients was 0.84±0.37, which was higher than that of the healthy controls (0.49±0.18) (*p* < 0.001).

The distribution of IgA-gH/gL levels according to the individual patient's cancer stage is shown in Figure 1B. We found that the median IgA-gH/gL OD value for patients with stage IV NPC (0.96±0.35) was higher than that of early stage (I+II) (0.81±0.28) and stage III patients (0.79±0.35), but this was not statistically significant (I+II *vs*. III *p* = 0.284, I+II *vs*. IV *p* = 0.204, III *vs*. IV *p* = 0.671). Additionally, we did not observe correlations between antibody level and other patient clinical characteristics, such as age, gender, smoking history and IgA-VCA or EBV DNA status (Table 1).

**Table 1:** Associations of EBV IgA-gH/gL and NPC patient clinicopathological parameters in the training cohort

Parameters	OD Value			No.	OD≥0.63		OD<0.63		P^a^
	Maximum	Minimum	Median		No.	(%)	No.	(%)	
Age, years									0.237
<46	2.317	0.27	0.85	111	96	86.5	15	13.5	
≥46	2.648	0.383	0.823	97	78	80.4	19	19.6	
Sex									0.674
Male	2.648	0.27	0.823	153	127	83	26	17	
Female	2.317	0.451	0.853	55	47	85.5	8	14.5	
Smoking history									0.713
Yes	2.648	0.383	0.805	98	81	82.7	17	17.3	
No	2.317	0.27	0.864	110	93	84.5	17	15.5	
Tumor stage									0.401
I+II	1.893	0.413	0.807	44	34	77.3	10	22.7	
III	2.648	0.27	0.793	110	93	84.5	17	15.5	
IV	2.381	0.529	0.957	54	47	87	7	13	
VCA									0.358
Positive	2.648	0.27	0.85	176	149	84.7	27	15.3	
Negative	2.317	0.437	0.807	32	25	78.1	7	21.9	
EBV DNA									0.327
Positive	2.648	0.27	0.869	149	127	85.2	22	14.8	
Negative	1.893	0.383	0.788	59	47	79.7	12	20.3	

OD, optical density at 450 nm; VCA, immunofluorescence assay for IgA antibodies against viral capsid antigen; EBV DNA, qPCR test for plasma circulating EBV DNA.

The OD value of 0.63 was the cut-off in the IgA-gH/gL ELISA test; a titer ≥1/40 was considered to be a positive result in the IgA-VCA IFA test; a detection of 100 copies/mL was chosen as the cut-off level for the EBV DNA test.

a Chi-squared test.

### Diagnostic values of IgA-gH/gL for NPC in the training cohort

**Figure 2:**
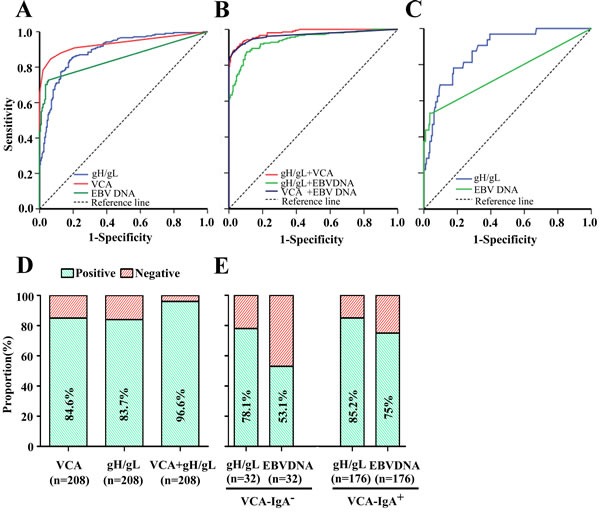
Diagnostic outcomes of gH/gL, VCA, EBV DNA and their combinations for detection of NPC in the training cohort ROC curve for gH/gL, VCA or EBV DNA for NPC patients *vs*. controls **A.** ROC curves for the combination of gH/gL and VCA, gH/gL and EBV DNA, or VCA and EBV DNA for NPC patients *vs*. controls **B.** ROC analyses of antibody responses to gH/gL or plasma EBV DNA concentrations in VCA-negative NPC patients, showing an AUC of 0.879 (95% CI, 0.820-0.937) for IgA-gH/gL and of 0.750 (95% CI, 0.637-0.863) for EBV DNA **C.** Rates of correct NPC diagnosis for VCA, gH/gL or both in NPC patients **D.** Rates of correct NPC diagnosis for gH/gL and EBV DNA in VCA-negative or VCA-positive patients **E.**

IgA-gH/gL was evaluated as a potential marker for the diagnosis of NPC using ROC (receiver operating characteristic) analysis based on OD values (Figure 2A, Table 2). Using a cut-off OD value of 0.63 for the gH/gL test, the IgA-gH/gL ELISA had a sensitivity of 83.7%, specificity of 82.3%, positive predictive value (PPV) of 83.3%, negative predictive value (NPV) of 82.7%, and AUC (area under the curve) of 0.893 (95% CI, 0.862-0.924). Compared to IgA-gH/gL, the IgA-VCA IFA had a similar sensitivity of 84.6% (*p* = 0.89), higher specificity of 93.4% (*p* = 0.002) and similar AUC of 0.933 (95% CI, 0.906 - 0.959) (*p* = 0.053). However, circulating EBV DNA had the lowest sensitivity at 71.6% (p = 0.004), AUC of 0.849 (95% CI, 0.810 - 0.889) (*p* = 0.046) and highest specificity of 94.9% (*p* < 0.001). These results showed that the diagnostic capacity of gH/gL was comparable to that of the other two EBV markers.

**Table 2:** AUC, sensitivity, specificity, PPV and NPV of IgA-gH/gL, IgA-VCA, EBV DNA and their combinations for detection of NPC in the training cohort

Test	AUC (95%)	p*^a^*	Sensitivity		p*^b^*	Specificity		p*^b^*	PPV		p*^c^*	NPV		p*^c^*
			%	95% CI		%	95% CI		%	95% CI		%	95% CI	
gH/gL	0.893 (0.862-0.924)		83.7	78.7-88.7		82.3	77.0-87.6		83.3	78.2-88.4		82.7	77.4-88.0	
VCA	0.933 (0.906-0.959)		84.6	79.7-89.5		93.4	89.9-96.9		93.1	89.5-96.7		85.3	80.6-90.0	
EBV DNA	0.849 (0.810-0.889)		71.6	65.5-77.7		94.9	91.8-98.0		93.7	89.9-97.5		76.1	70.8-81.4	
gH/gL vs. VCA		NS			NS			0.002			0.003			NS
gH/gL vs. EBVDNA		0.046			0.004			<0.001			0.002			NS
VCA vs. EBVDNA		<0.001			0.001			NS			NS			0.013
gH/gL + VCA	0.980 (0.970-0.991)		88.5	84.2-92.8		97.0	94.6-99.4		96.8	94.3-99.3		88.9	84.7-93.1	
gH/gL + EBV DNA	0.941 (0.919-0.963)		87.5	83.0-92.0		89.4	85.1-93.7		89.7	83.5-93.9		87.2	82.6-91.8	
VCA + EBV DNA	0.967 (0.949-0.985)		87.5	83.0-92.0		97.4	95.2-99.6		97.3	95.0-99.6		88.1	83.8-92.4	
gH/gL vs.gH/gL + VCA		<0.001			NS			<0.001			<0.001			NS
VCA vs.gH/gL + VCA		<0.001			0.039			NS			NS			NS
EBV DNA vs.gH/gL + VCA		<0.001			<0.001			NS			NS			<0.001
gH/gL + VCA vs. gH/gL + EBV DNA		<0.001			NS			0.001			0.005			NS
gH/gL + VCA vs. VCA + EBV DNA		NS			NS			NS			NS			NS

PPV, positive predictive value; NPV, negative predictive value; NS, not significant; gH/gL, enzyme-linked immunosorbent assay detection of IgA antibodies against EBV glycoprotein complex gH/gL; VCA, immunofluorescence assay for IgA antibodies against viral capsid antigen; EBV DNA, qPCR test for plasma circulating EBV DNA.

a Z test.

b McNemar's paired-sample test.

c Chi-squared test.

### Establishment of a logistic regression model combining IgA-gH/gL and IgA-VCA

Because IgA-gH/gL alone did not show an advantage in the detection of NPC in comparison to IgA-VCA, we used a binary logistic regression model to assess whether a combination of EBV markers could improve diagnostic efficiency (Figure 2B, Table 2). The combination of IgA-gH/gL and IgA-VCA achieved similar sensitivity (88.5%), specificity (97%), PPV (96.8%) and NPV (88.9%) and an AUC of 0.980 (95% CI, 0.970 - 0.991) as the combination of IgA-VCA and EBV DNA (87.5%, 97.4%, 97.3%, 88.1%, and 0.967 [95% CI, 0.949 - 0.985], respectively), which was superior to the combination of IgA-gH/gL and EBV DNA (87.5%, 89.4%, 89.7%, 87.2%, and 0.941 [95% CI, 0.919 - 0.963], respectively). We selected the logistic regression model that combines IgA-VCA with the IgA-gH/gL ELISA as the new optimal combination for NPC detection. The following formula was established:

$$\mathrm{Log}\frac{p}{\left(1-p\right)}=-7.36+0.055\times \text{VCA}+8.133\times \text{gH}/\text{gL}$$

where VCA represents the reciprocal transformation of the titer of IgA to VCA and gH/gL represents the OD at a wave length of 450 nm of IgA to gH/gL.

In this model, the ROC analysis (Figure 2B) showed that testing of both VCA and gH/gL increased the NPC diagnostic capacity (AUC, 0.98; 95% CI, 0.97 - 0.991) compared to either individual test. A total of 201 (96.6%) of the 208 NPC patients had positive results when VCA and gH/gL were tested together (Figure 2D). Furthermore, 25 (78.1%) of 32 VCA-negative patients with NPC had positive gH/gL results (Figure 2E). The rate was similar (150 [85.2%] of 176) to that observed in the VCA-positive patients (Figure 2E). Fifteen IgA-VCA-negative NPC patients were at stage I or II and the rate of positive gH/gL among them was 66.7%. Seventeen IgA-VCA-negative NPC patients were at stage III or IV and the rate of positive gH/gL among them was 88.2%. However, we did not find any significant difference among different clinical stages (*p* = 0.337). The ROC curves for gH/gL indicated a diagnosis of NPC in patients with negative VCA (Figure 2C), with a sensitivity of 78.1% and AUC of 0.879 (95% CI, 0.820 - 0.937). In the case of EBV DNA, the most specific assay with the highest positive predictive value was the combination of IgA-VCA and EBV DNA, but only 17 (53.1%) of the 32 VCA-negative patients with NPC had positive EBV DNA results (Figure 2E). This rate was lower than that of the VCA-positive patients (132 [75%] of 176). EBV DNA had a sensitivity of 53.1% and AUC of 0.750 (95% CI, 0.637 - 0.863); these values were inferior to those of gH/gL (Table 3).

**Table 3:** AUC, sensitivity, specificity, PPV and NPV of IgA-gH/gL and EBV DNA in IgA-VCA-negative NPC patients in the training cohort

Test	AUC (95%)	p*^a^*	Sensitivity		p*^b^*	Specificity		p*^b^*	PPV		p*^c^*	NPV		p^c^
			%	95% CI		%	95% CI		%	95% CI		%	95% CI	
gH/gL	0.879 (0.820-0.937)		78.1	63.6-92.4		82.3	77.0-87.6		42.0	29.5-54.5		96.0	93.0-99.0	
EBV DNA	0.750 (0.637-0.863)		53.1	35.8-70.4		94.9	91.8-98.0		63.0	44.8-81.2		92.6	89.0-96.2	
gH/gL vs. EBV DNA		0.003			0.021			<0.001			NS			NS

PPV, positive predictive value; NPV, negative predictive value; NS, not significant; gH/gL, enzyme-linked immunosorbent assay detection of IgA antibodies against EBV glycoprotein complex gH/gL; VCA, immunofluorescence assay for IgA antibodies against viral capsid antigen; EBV DNA, qPCR test for plasma circulating EBV DNA.

a Z test.

b McNemar's paired-sample test.

c Chi-squared test

### Validating the logistic regression model combining IgA-gH/gL and IgA-VCA

**Figure 3:**
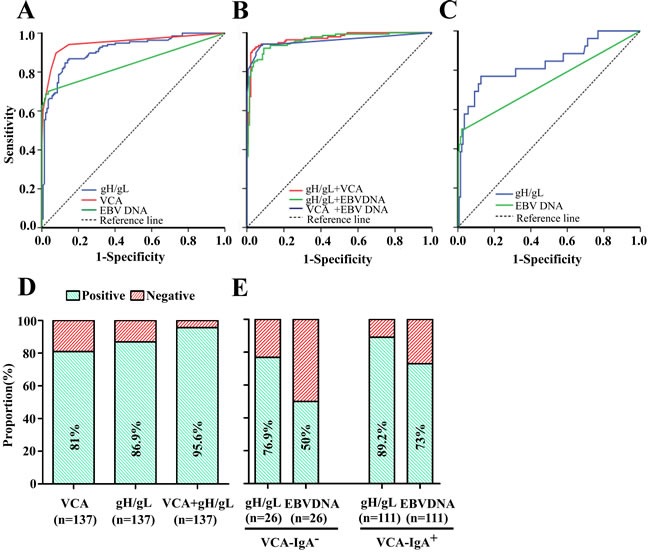
Diagnostic outcomes of gH/gL, VCA, EBV DNA and their combinations for detection of NPC in the validation cohort ROC curve for gH/gL, VCA or EBV DNA for NPC patients *vs*. controls **A.** ROC curves for the combination of gH/gL and VCA, gH/gL and EBV DNA or VCA and EBV DNA NPC patients *vs*. controls **B.** ROC analyses of antibody responses to gH/gL or plasma EBV DNA concentrations in VCA-negative NPC patients, showing an AUC of 0.835 (95% CI, 0.737-0.934) for IgA-gH/gL and of 0.740 (95% CI, 0.612-0.867) for EBV DNA **C.** Rates of correct NPC diagnosis for VCA, gH/gL or both in NPC patients **D.** Rates of correct NPC diagnosis for gH/gL and EBV DNA in VCA-negative or VCA-positive patients **E.**

To further verify the efficacy of the regression model estimated from the training data set, we applied it in an independent validation cohort to diagnosis NPC (*n* = 137 patients with NPC and 142 healthy controls). With a cut-off OD value of 0.63 for the IgA-gH/gL alone, we observed similar results in the validation cohort as in the training cohort (Figure 3A, Table 4). IgA-gH/gL ELISA had good diagnostic capacity with a sensitivity of 86.9%, specificity of 80.3%, positive predictive value (PPV) of 81%, negative predictive value (NPV) of 86.3%, and AUC of 0.912 (95% CI, 0.878 - 0.947). Additionally, the validation cohort confirmed the ability of IgA-gH/gL to diagnose NPC in VCA-negative patients, with a sensitivity of 76.9% and AUC of 0.835 (95% CI, 0.737 - 0.934) (Table 5, Figure 3C). Twenty (76.9%) of 26 VCA-negative patients with NPC had positive gH/gL results (Figure 3E). This rate was similar to that observed in the VCA-positive patients (99 [89.2%] of 111) (Figure 3E). Nine IgA-VCA-negative NPC patients were at stage I or II and the rate of positive gH/gL among them was 88.9%. The improvement in NPC diagnosis resulting from measurement of IgA-gH/gL and IgA-VCA together was also observed in the validation cohort (Table 4, Figure 3B). ROC analysis showed that the newly established regression model had a sensitivity of 91.2%, specificity of 96.5%, PPV of 96.2%, NPV of 91.9%, and AUC of 0.97 (95% CI, 0.951 - 0.988). A total of 131 (95.6%) of 137 NPC patients were diagnosed correctly when VCA and gH/gL were tested together (Figure 3D).

**Table 4:** AUC, sensitivity, specificity, PPV and NPV of IgA-gH/gL, IgA-VCA, EBV DNA and their combinations for detection of NPC in the validation cohort

Test	AUC (95%)	p*^a^*	Sensitivity		p*^b^*	Specificity		p*^b^*	PPV		p*^c^*	NPV		p*^c^*
			%	95% CI		%	95% CI		%	95% CI		%	95% CI	
gH/gL	0.912 (0.878-0.947)		86.9	81.3-92.5		80.3	73.8-86.8		81.0	74.7-87.3		86.3	80.4-92.2	
VCA	0.950 (0.922-0.977)		81.0	74.4-87.6		95.0	91.0-98.6		94.1	89.8-98.4		83.9	78.2-89.6	
EBV DNA	0.844 (0.794-0.893)		69.3	61.6-77.0		96.5	93.5-99.5		95.0	90.7-99.3		76.5	70.3-82.7	
gH/gL vs. VCA		NS			NS			<0.001			0.002			NS
gH/gL vs. EBVDNA		0.009			<0.001			<0.001			0.001			NS
VCA vs. EBVDNA		<0.001			0.015			NS			NS			NS
gH/gL + VCA	0.970 (0.951-0.988)		91.2	86.5-95.9		96.5	93.5-99.5		96.2	92.9-99.5		91.9	87.5-96.3	
gH/gL + EBV DNA	0.961 (0.940-0.982)		89.0	83.8-94.2		91.5	86.9-96.1		91.0	86.2-95.9		89.7	84.8-94.6	
VCA + EBV DNA	0.959 (0.933-0.985)		81.0	74.4-87.6		99.3	97.9-100.0		99.0	97.2-100.0		84.4	78.9-89.9	
gH/gL vs.gH/gL + VCA		<0.001			NS			<0.001			<0.001			NS
VCA vs.gH/gL + VCA		NS			0.001			NS			NS			0.03
EBV DNA vs.gH/gL + VCA		<0.001			<0.001			NS			NS			<0.001
gH/gL + VCA vs. gH/gL + EBV DNA		NS			NS			NS			NS			NS
gH/gL + VCA vs. VCA + EBV DNA		NS			0.011			NS			NS			0.04

PPV, positive predictive value; NPV, negative predictive value; NS, not significant; gH/gL, enzyme-linked immunosorbent assay detection of IgA antibodies against EBV glycoprotein complex gH/gL; VCA, immunofluorescence assay for IgA antibodies against viral capsid antigen; EBV DNA, qPCR test for plasma circulating EBV DNA.

a Z test.

b McNemar's paired-sample test.

c Chi-squared test.

**Table 5:** AUC, sensitivity, specificity, PPV and NPV of IgA-gH/gL and EBV DNA in IgA-VCA-negative NPC patients in the validation cohort

Test	AUC (95%)	p*^a^*	Sensitivity		p*^b^*	Specificity		p*^b^*	PPV		p*^c^*	NPV		p*^c^*
			%	95% CI		%	95% CI		%	95% CI		%	95% CI	
gH/gL	0.835 (0.737-0.934)		76.9	60.7-93.1		80.3	73.8-86.9		41.7	27.8-55.6		95.0	91.1-98.9	
EBV DNA	0.740 (0.612-0.867)		50.0	30.8-69.2		96.5	93.5-99.5		72.2	51.5-92.9		91.3	86.8-95.8	
gH/gL vs. EBV DNA		NS			NS			<0.001			0.027			NS

PPV, positive predictive value; NPV, negative predictive value; NS, not significant; gH/gL, enzyme-linked immunosorbent assay detection of IgA antibodies against EBV glycoprotein complex gH/gL; VCA, immunofluorescence assay for IgA antibodies against viral capsid antigen; EBV DNA, qPCR test for plasma circulating EBV DNA.

a Z test.

b McNemar's paired-sample test.

c Chi-squared test.

## DISCUSSION

In this study, we found that IgA-gH/gL had a similar sensitivity for the diagnosis of NPC compared with IgA-VCA IFA (*p* = 0.89), but was more sensitive than plasma EBV DNA (*p* = 0.004). IgA-gH/gL had an AUC of 0.893 (95% CI, 0.862-0.924), which was better than that of EBV DNA (*p* < 0.05). The combination of IgA-gH/gL and IgA-VCA could improve the detection of NPC in patients.

We chose IgA-VCA as the reference marker because it has been widely used in clinical diagnosis and population screening studies due to its relatively high sensitivity and specificity. However, the EBV serological spectrum differs among individuals, and 4 - 24% of patients with NPC remain negative for VCA [6, 9–12]. Consequently, a combination of different markers, especially novel markers with high diagnostic capacity among VCA-negative patients, is extremely important for diagnostic accuracy. A combination of circulating EBV DNA and IgA-VCA, studied by Leung, *et al.* [17], showed that seven NPC patients who had false negative results for EBV DNA did not overlap with the 27 NPC patients who had false negative results for IgA-VCA, except in two cases. Similarly, Dardari, *et al.* [18] reported that 89% of sera from young Maghrebian NPC patients that were negative for IgA-VCA were positive at very high titers for IgG antibodies to the EBV transactivator protein (ZEBRA). More recently, positive IgA/VCA-p40+18 antibody ELISA results in 63.6% of nasopharyngeal carcinoma samples were missed entirely by peptide-based IgA/EBV-ELISAs (peptides derived from immunodominant epitopes of EBNA1 and VCA-p18) [19].

In our report, the sensitivity of IgA-gH/gL was close to that of IgA-VCA. Of note, 15.4% of all NPC patients in the training cohort were IgA-VCA negative; however, gH/gL maintained diagnostic capacity (25 of the 32 VCA-negative NPC patients had positive gH/gL results), and 201 (96.6%) of the 208 NPC patients were diagnosed correctly when VCA and gH/gL were tested together. The logistic regression analysis showed that IgA-VCA combined with IgA-gH/gL ELISA was an effective method for differentiating between NPC patients and controls, and these results were validated in a second independent cohort. Therefore, IgA-gH/gL detection complemented the IgA-VCA test for NPC diagnosis.

Plasma EBV DNA, which is another new non-protein serum marker for NPC screening, has also been reported to perform well in diagnostic tests [17, 20]. In our study, EBV DNA had the highest specificity (94.9%) and positive predictive value (93.7%) among the three biomarkers. The combination of EBV DNA and IgA-VCA showed excellent diagnostic performance, especially in specificity (97.4%) and positive predictive value (97.3%). However, EBV DNA might be more attractive as an independent prognostic factor for relapse risk or overall patient survival, because DNA load corresponded to NPC progression and remission [21].

EBV gH, also known as gp85, is an EBV membrane antigen encoded by the EBV late lytic gene BXLF2, which corresponds to the EBV lytic-cycle product [4]. The gH and gL proteins associate to form a heterodimer necessary for efficient membrane fusion [22]. Khanna, *et al.* [23] demonstrated that cytotoxic t-lymphocytes (CTLs) from acute infectious mononucleosis patients displayed strong *ex vivo* reactivity against EBV gH, and suggested that CTL epitopes from EBV gH could be exploited in the development of an EBV vaccine. Urquiza, *et al.* [24] found that peptide 11438 from the EBV gH host cell-binding region inhibited EBV infection of peripheral blood mononuclear cells (PBMCs). The peptide induced production of antibodies that recognize EBV and EBV-infected cells.

Additional studies have associated a host immune response with other types of herpesvirus gH. For example, varicella-zoster virus gH/gL is reactive with the anti-human gamma chain of IgG near the glycosylation site [25], and baculovirus expression of recombinant chelonid fibropapillomatosis-associated herpesvirus (CFPHV) gH has been successfully applied to detect turtle antibodies to recombinant CFPHV gH using ELISA [26]. The application of EBV glycoproteins in NPC screening has also been previously reported. gp350/220 induces a strong immune response in EBV-infected individuals, and several studies have detected IgA-gp350/220 in NPC patients at higher titers than in healthy controls [27, 28]. Although the function of gp78 remains unknown, the sensitivities and specificities of IgA-gp78 and IgG-gp78 have both exceeded 70% as reported by Gu, *et al.* [29]. The VCA complex protein, the antigen most commonly used for serological diagnosis, is composed of several different proteins. One of these, gB (gp125), is believed to be a dominant immunogen of the VCA complex [30, 31], and several ELISA kits based on VCA-gp125 have been commercialized for NPC [6].

Our study had several limitations. The sample size was small, and to confirm the efficacy of IgA-gH/gL, a multi-center study is required. Additionally, detection of IgA antibodies against the EBV early antigen in sera was demonstrated to be a strong indicator of NPC [6, 9]; whether its combination with IgA-gH/gL could achieve a better diagnostic outcome warrants further exploration.

## MATERIALS AND METHODS

### Study population

A total of 208 serum samples in a training cohort were collected from histologically confirmed NPC patients before treatment. All patients were histologically diagnosed with WHO type II or III NPC [32] at the Sun Yat-sen University Cancer Center (SYSUCC), and had definite clinical stage NPC according to the seventh American Joint Committee on Cancer TNM staging manual [33]. We randomly selected 198 healthy controls, including 138 males and 60 females ranging in age from 30 to 60 years (mean age = 44.1 years), from the hospital staff who participated in the medical examination projects. A validation cohort comprising 137 patients with NPC and 142 healthy controls was also recruited from SYSUCC. We matched the groups in the two cohorts for age and sex as best as possible. The characteristics of the patients are presented in the Table S1. This blinded study was approved by the Clinical Research Ethics Committee of the SYSUCC, and all of the participants provided written informed consent.

### Cell culture

High Five^TM^ insect cells (a gift from Yun Wang, Wuhan Institute of Virology, Chinese Academy of Sciences, Hubei, China, wangyun@wh.iov.cn) were used for protein expression and were grown in shaker flasks in Express Five serum-free medium (Invitrogen, Grand Island, N Y). Sf9 insect cells used for baculovirus production were grown in 150-cm^2^T flasks in Sf-900 II serum-free medium (Invitrogen). All media contained penicillin-streptomycin and amphotericin B (Sigma-Aldrich, St Louis, MO, USA).

### EBV gH, EBV gL constructs

P2089 EBV plasmid (gift from Wolfgang Hammerschmidt, German Research Center for Environmental Health, Munich, Germany, hammerschmidt@helmholtz-muenchen.de) was used as the template to PCR-amplify EBV gH/gL. The construction of soluble EBV gH/gL has been described previously [14]. In the expression constructs, gH (aa 18-679) [GenBank: NC009334] and gL (aa 24-137) [GenBank: YP001129472] were fused to the baculovirus gp64 signal sequence, and a flag epitope tag was inserted into the carboxyl termini of each protein. The related primers and gp64 are listed in Table S2. pFastBacHTB and DH10Bac^TM^ *E. coli* were kindly provided by Yun Wang.

### Protein expression and purification

One liter of High Five^TM^ insect cells at a density of 2×10^6^ cells/mL in shaker flasks was infected with the P3 gH/gL baculovirus at 27°C with stirring at 100 rpm. Supernatants were harvested at 72 h post-infection under the optimal experimental conditions. The supernatants were concentrated and sterile filtered. The expressed six-His-tagged protein was purified from supernatants by Ni chelate-Sepharose (Qiagen-Sample & Assay Technologies, Hilden, Germany) chromatography according to the manufacturer's protocol. After washing the combined agarose with wash buffer (50 mM NaH_2_PO_4_, 300 mM NaCl, 20 mM imidazole and pH 8.0), purified recombinant proteins were eluted with elution buffer (50 mM NaH2PO4, 300 mM NaCl, 300 mM imidazole and pH 7.5). Eluted proteins were exchanged into PBS by ultrafiltration. Purified proteins were concentrated 10-fold, dialyzed and stored at −80°C.

### SDS/PAGE and western blot analysis of the EBV gH/gL

Ten μL of eluted protein was denatured in 10 μL 2x sodium dodecyl sulfate polyacrylamide gel electrophoresis (SDS/PAGE) loading buffer (0.2% Bromophenol Blue, 20% (v/v) glycerol, 4% (w/v) SDS, 100 mmol/l Tris/HCl, pH 6.8, and 200 mmol/L dithiothreitol) by boiling at 98°C for 10 min. The samples were subjected to 10.5% acrylamide resolving gels, stained with Coomassie Blue for at least 30 min and then destained in methanol-acetic acid. Proteins were transferred onto PVDF membranes at 200 mA for 3 h. The membranes were blocked for 45 min at room temperature with 5% nonfat milk in PBS-T buffer (containing 0.1% Tween 20). Membranes were then incubated with primary antibody (1:1000; anti-flag tag mouse polyclonal antibody, flag-M2, Sigma-Aldrich) at 4°C overnight, followed by secondary antibody (anti-mouse IgG) coupled to horseradish peroxidase (HRP) for at least 45 min.

### ELISA detection of antibodies to recombinant gH/gL

The purified recombinant gH/gL was dissolved in 10 mM PBS at pH 7.4. The 96-well microtiter plates were coated with 450 ng of gH/gL protein per well at 37°C for 2 h. After incubation, unoccupied sites were blocked with 3% BSA in PBS. The plates were washed five times with PBS containing 0.1% Tween-20 (PBS-T). After washing, serum samples (1:100 in PBST containing 3% BSA) were added and incubated for 1 h at 37°C. After five washes, HRP-labelled goat anti-human IgA antibodies (1:6000; Boster corporation, Wuhan, China) for gH/gL in PBS-T were added. The plates were incubated at 37°C for 30 min and washed before adding a tetramethylbenzidine reagent (Sigma-Aldrich, St Louis, MO, USA) for 10 min at 37°C. The reaction was stopped with 2 M H_2_SO_4_ and the optical density (OD) at 450 nm was determined using an ELISA reader.

### IgA-VCA immunofluorescence assay and plasma EBV assay

The EBV-specific IgA-VCA antibody was assessed using a previously described immunofluorescent method [2, 6]; a titer ≥1/40 was considered to be positive. The procedures for real-time quantitative PCR (RT-qPCR) and measurement of plasma EBV DNA were described in previous studies [11, 34]. The RT-qPCR system was developed for plasma EBV DNA detection toward the BamHI-W region. The system consisted of the amplification primers W-44F (5′-AGT CTC TGC CTC AGG GCA-3′) and W-119R (5′-ACA GAG GGC CTG TCC ACCG-3′) and the dual-labeled fluorescent probe W-67T (5′-[FAM] CAC TGT CTG TAA AGT CCA GCC TCC [TAMRA]-3′). A detection level of 100 copies/mL was chosen as the cut-off level.

### Statistical analysis

The results were analyzed using the statistical software SPSS for Windows (version 16.0), MedCalc (version 13.0.2.0) and GraphPad Prism (version 5.0). Participants’ characteristics and NPC risk factors were compared using chi-squared tests. The Mann-Whitney U test was used to analyze the differences between patients with NPC and healthy controls. ROC curves were constructed to assess sensitivity, specificity and area under the curve (AUCs) using 95% CI. The cut-off value for the biomarker was defined as the value with the highest sensitivity and specificity selected from the respective ROCs [35]. Chi-squared tests were used to compare the mean IgA-gH/gL OD among patients at different cancer stages. McNemar's paired-sample test or chi-squared test was applied to determine whether the sensitivity, specificity, positive predictive values and negative predictive values of gH/gL, VCA, EBV DNA and their combinations were significantly different, and AUC comparisons were assessed using Z tests. Binary logistic regression models of all possible combinations of biomarkers were used to select the optimal combination for NPC diagnosis. *P* < 0.05 was considered to indicate statistical significance, and all of the statistical tests were two-sided.

## SUPPLEMENTARY MATERIAL FIGURE AND TABLES


