# Supplementary material for: Epstein-Barr virus glycoprotein gH/gL antibodies complement IgA-viral capsid antigen for diagnosis of nasopharyngeal carcinoma
Source: Oncotarget. 2016 Feb 24;7(13):16372–83. doi: 10.18632/oncotarget.7688 (PMC4941321; doi:10.18632/oncotarget.7688)
Supplement: Supplementary file 1 [file oncotarget-07-16372-s001.pdf]

Epstein-Barr virus glycoprotein gH/gL antibodies complement IgA-viral capsid antigen for diagnosis of nasopharyngeal carcinoma

Supplementary Material

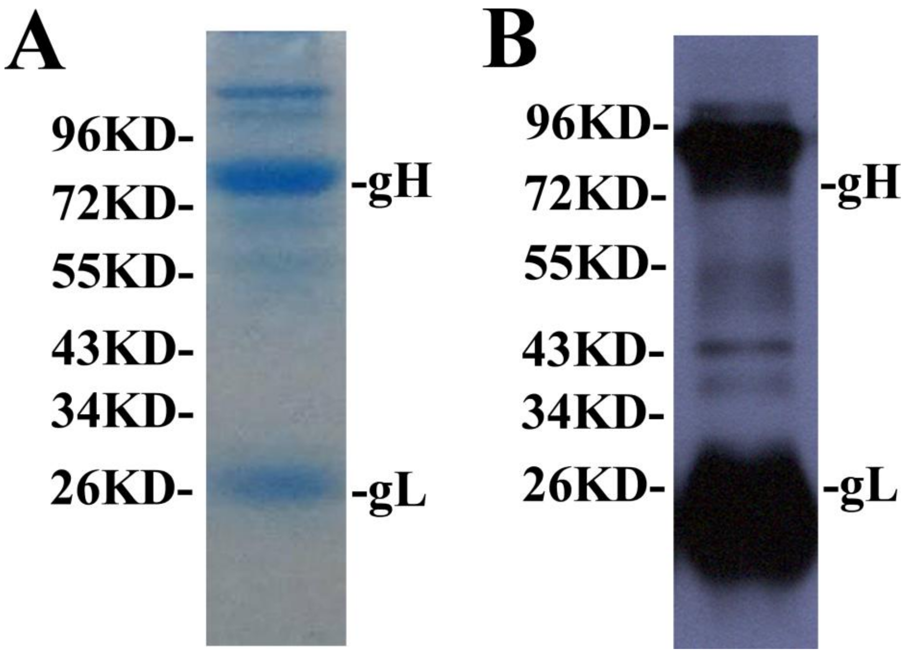

Supplementary Figure 1: Production and purification of soluble EBV gH/gL proteins. SDS-PAGE showing gel filtration-purified samples of soluble gH/gL **A.** Western blotting of the recombinant protein identified by anti-Flag antibody **B.** The values on the left are molecular sizes in kilodaltons.

Supplemental Table 1: Clinical characteristics of NPC patients and healthy controls.

Characteristic	Training					Validation				
	NPC (208)		Controls (198)		P[a]	NPC (137)		Controls (142)		P[a]
	No.	%	No.	%		No.	%	No.	%	
Age, years					0.973					0.638
<46	111	53.4	106	53.5		81	59.1	80	56.3	
≥46	97	46.6	92	46.5		56	40.9	62	43.7	
Sex					0.388					0.152
Male	153	74	138	70		105	76.6	98	69	
Female	55	26	60	30		32	23.4	44	31	
Family history of NPC					<0.001					0.009
Yes	27	13	6	3		11	8	2	1.4	
No	181	87	192	97		126	92	140	98.6	
Smoking history					0.662					0.626
Yes	98	47	89	45		84	61.3	83	58.5	
No	110	53	109	55		53	38.7	59	41.5	
IgA-VCA					<0.001					<0.001
Positive	176	85	13	7		111	81	7	5	
Negative	32	15	176	93		26	19	135	95	
Tumor stage										

I	10	4.8	5	3.7
II	34	16.3	8	5.8
III	110	52.9	63	46
IV	54	26	61	44.5

IgA-VCA, immunofluorescence assay for IgA antibodies against viral capsid antigen.

A titer≥1/40 was taken as a positive result in the IgA-VCA IFA test.

[a] Chi-square test.

Supplemental Table 2: Primers and gp64 signal sequence.

Primer	sequence
gH	
Sense	5' GA AGATCT GCCAGTCTCAGCGAGGTTAAG 3'
Antisense	5' CCGCTCGAGTTAAAGCTTGTCGTCATCGTCTTTGTAGTC GTGTGCTCTTTCTTCATAC 3'
gL	
Sense	5' GA AGATCT TGGGCATACCCATGTTGTCACG 3'
Antisense	5' CCGCTCGAGTTAAAGCTTGTCGTCATCGTCTTTGTAGTC GCCCCCGCGATGCCATGC 3'
pUC/M13	
Sense	5’ CCCAGTCACGACGTTGTAAAACG 3’
Antisense	5’ AGCGGATAACAATTTCACACAGG 3’

gp64 signal sequence

ATGGTAAGCGCTATTGTTTTATATGTGCTTTTGGCGGCGGCGGCGCATTCTGCCTTTGCG
